# Opening Address

**Published:** 1846-03

**Authors:** Robert Arthur


### ARTICLE II.

*Opening Address.* Delivered before the Pennsylvania Association of Dental Surgeons, January 20th, 1846. By Robert Arthur, D. D. S. Published by order of the Society.

Gentlemen:

A few weeks since, after we had the pleasure of effecting the formation of an association of practitioners of our profession in this state, you requested me to deliver before you the opening address, and I immediately consented to do so. Upon reflection, however, I almost regretted that I had given my consent to make an attempt, for which my habits render me so little fitted; and I take this stand, in the fulfilment of the duty you have imposed upon me, with as keen a sense of the peculiar delicacy of my position, as any one present. To nearly all of you I am an entire stranger, and, in age and professional experience, am the junior of many whom I see before me; in such circumstances, is it possible for me to divest myself entirely of fear of the charge of presumption, in undertaking what I cannot but regard as an important duty. It has, indeed, seemed to me that this task should properly have been imposed upon an older and more experienced member of this society—one with whom you were more familiar, and in whose judgment you could have more confidence than it is possible you can have in mine. I should have been glad of a release from this engagement, did it not furnish me with an occasion to say some things which I am disposed to believe may have a useful tendency. And, since you have been pleased to select me for the purpose, I sincerely hope, that what I am about to say may meet with your approbation, and contribute, in some slight degree, towards the more perfect accomplishment of the important objects which brought us together.

Upon this occasion, many interesting topics present themselves, to which, if time permitted, I would gladly give attention; but I shall be compelled to confine myself to those which seem of first importance. Of these, there are two which appear to me most appropriate, and which I shall endeavor to bring to your notice. If, in my presentation of them, you should not find any thing striking or useful, I will at least hope that they may direct your thoughts in a channel which will lead to something useful.

I will endeavor to engage your attention, then, with a discussion of

1st. *The objects hoped to be gained by means of the association into which we have just entered; and*

2dly. *The ends which we should keep steadily in view, in order to insure the permanent success of our efforts towards the accomplishment of these objects.*

With regard to the objects of this association, it was my intention to have said little, as they seemed to me to be too obvious to require any effort to impress them upon you. But a remark which fell from a gentleman, between whom and myself a few words of conversation passed relative to the subject, to the effect that he did not see any great good which could result from an association of this kind, has led me to believe that there was not so general an appreciation of the benefits of association, as I had at first supposed, and has induced me to attempt to show how a society such as ours, constituted upon correct principles, and steadily supported, with right ends in view, must be of incalculable benefit to us all, and, what is of much more importance, tend greatly to increase our usefulness in the community.

The dental profession, as a distinct profession, is just beginning to be recognized by the public. A few years ago, it was very generally believed, by those who had any kind of confidence in the treatment of diseases of the teeth, that neither intelligence nor special education for the practice of dental surgery was necessary; all that was believed to be required, was a certain amount of mechanical ingenuity. Many, therefore, who found themselves unsuccessful in other employments, or conceived a notion that operating upon the teeth was more lucrative than any other vocation in which they could engage, as it required no outlay of time or money for preparation, called themselves dentists, and were received, countenanced and sustained as such, by the public. The consequence of this state of things was, that a number of unqualified persons, far greater than those who were competent to practice it, were introduced into the profession, and thus a great obstacle thrown in the way of its advancement. So great, indeed, and common were the injuries inflicted by this class of persons, that many, who were willing and anxious to believe that dental surgery had important resources, were compelled to come to the conclusion that the whole pretended science was an imposture, and that dentists always did more harm than good by their attentions to the teeth. At the time to which I am now alluding, there were very few, indeed, of the many engaged in it, who had any claims to a position in so important a profession, for the means of instruction were scanty, and hard to be reached. Although a very few years brought about a considerable change in the character of the profession, and the community began to be convinced of its usefulness, it still remained open for all who thought proper to claim its privileges, and many improper persons continued to thrust themselves forward. To those who had labored hard and faithfully, to render themselves competent to perform the duties which their station imposed upon them, it was, naturally, a source of chagrin to witness the effects of the efforts of these charlatans, to be classed with them, and to share the disgrace which, through their agency, was brought upon their vocation. As this class was very large, and as there were no means of judging of the claims which many of those who presented themselves had to their respect, it was impossible that there could be any thing like free intercourse amongst those practising the profession—each was rendered suspicious of the other. In this way, it was quite natural that, in the endeavor to shun the improper, those who were worthy of all esteem should be overlooked; and thus has a spirit of the most chilling reserve grown up, which has extended itself throughout the profession, and, in a greater or less degree, affected all its members. Now, although we well know that a very large proportion of those, even at this late day, presenting themselves for public patronage as practitioners of dental surgery, are altogether unworthy of confidence, we know, too, that there are also amongst them many estimable men, who are well qualified for the performance of the duties imposed upon them by their profession.

But how are we, now, to distinguish between the worthy and the unworthy, if we have no common plan upon which we can meet and learn to know each other? And what better plan can we find, for this purpose, than that of an association, having objects in view of great interests to all concerned in it, which will induce us to meet regularly together, and place us upon a pleasant social relation towards each other. Indeed, I am fully convinced, from the little experience we have thus far had, that it is only necessary for the members of the dental profession, in this city and state, to know each other more intimately, to find that there are many filling its ranks, who are worthy of the highest respect and esteem. Who that was present at the opening of the convention, called for the purpose of organizing this association, could have failed to observe the great coldness which prevailed at the meeting, but which gradually passed away, and, as liberal sentiments began to be poured forth, finding a ready response from men whom we had been almost disposed to look upon as narrow-minded, and regardless of any thing but their own selfish interests, but who were looking up steadily and earnestly, as ourselves, to the elevation of the profession; and the increase of its usefulness was replaced by a cheering warmth and a delightful sphere of good feeling. When we first met, we were all, or nearly all, strangers to each other; but we soon grew familiar, and in faces which were unknown before, we now see familiar features, and feel that many of these acquaintances may soon become friends. If we have made such rapid progress in the short intercourse we have had with each other, are we unwarrantable in believing that, by means of this association, we may soon attain to a higher, warmer and more elevating social professional intercourse with each other, than has ever yet existed among us. And this has seemed to me to be one of the principal objects of the Pennsylvania Association of Surgeon Dentists.

And if we are to hope for so much pleasure and profit from the improvement of our social relations towards each other, how much of incalculable value may we reasonably expect to gain from a free interchange of views, with regard to subjects strictly professional in their character. You will all be ready to admit the great advantages to be derived from common observation, directed to any particular subject; and it seems scarcely necessary to say any thing to establish what is so self-evident—it may be useful, however, for illustration, to show how common observations may be advantageously applied to our profession. For this purpose, I will for a little while direct your attention to an article which has, for a few years only, been used in the practice of dental surgery—in some cases very successfully, but in others, perhaps a majority, with injurious consequences. I allude, as you will perhaps already anticipate, to arsenic.

This article, as you are all, of course, aware, is used for the purpose of lessening the pain generally attendant upon dental operations, and in particular cases to effect a purpose which has always been very desirable, but which it has, heretofore, been difficult, if not impossible to accomplish. By many distinguished practitioners it has been pronounced, not only wholly useless for the purpose for which it is now extensively used, but positively injurious, and should be abandoned by every one who has at heart a desire to see his profession elevated, and wishes for the good of the community. With due deference to the opinion of these gentlemen, and the opinion of some of them, with regard to these subjects, I am not disposed to forget one entitled to the greatest degree of deference. I will take this occasion to state, that many who are rapidly and deservedly gaining a high rank in the profession, assert, positively, that very important results have followed the use of this article, in their hands. Amongst those who would, as yet, be very unwilling to lay aside what appears to them to be in the highest degree useful, I do not hesitate to class myself, and, until I can be convinced by actual facts, from which I can draw satisfactory conclusions, why it must act injuriously, I shall continue to use it. I do not, however, undertake to say that it may not exert an injurious influence—a few years ago I was convinced, from what I had observed of its effects, that it was a bad article, and had almost come to the conclusion to lay it aside. But subsequent experience has given me reason to believe, that the injurious results which I observed to follow the use of arsenic, were owing to the improper manner in which I made use of it. I now feel satisfied that it may eventually become a most important addition to the means we already have in our power of alleviating pain, although I think that we shall, for a long time, find it necessary to watch, closely, its effects, to reach the most safe and proper method of using it.

But, admit that it is improper for this purpose—how are we, who use it, to be satisfied of the fact. The results, which have followed the use of it, in our hands, have been very beneficial, and the experience of others, without we have the fullest knowledge of the particulars of the manner in which it was used, and the circumstances attendant upon the cases, cannot (especially when we must all be aware that observation of the effects of this article has been confined within very narrow limits) satisfy our minds. We must examine for ourselves.

Now it may be in the power of every member of the profession, to satisfy himself with regard to this and every other subject connected with it, and to arrive at correct conclusions, by his own unassisted efforts. But you will readily admit that he is much more liable to fall into error. The article to which I am now alluding, is strongly in point, for, as has been the case in a great many instances, if the first few experiments be made imperfectly or improperly, the results may be so bad as to deter the practitioner from pushing forward his investigations or bring him to the conclusion that he has nothing to gain from the use of arsenic, and induce him to throw it aside altogether.

Now, suppose the members of an association such as ours, were to determine to direct their attention to the investigation of this subject, and were to push their inquiries after a plan upon which they were all agreed, and were to meet at regular intervals and compare their experience, giving a detailed account of their manner of using the preparation, with all the peculiar circumstances attendant upon each case—the successes and failures. It will be at once seen, that if any errors were committed, they would be more likely to be discovered and corrected than if one individual had gone on alone. If there were any merit at all in the article for this purpose, success would be sure to follow the efforts of some—the cause of this would be investigated, and those who failed would be enabled to ascertain their errors and correct them.

Common observation directed to a particular subject, may be called concentrated experience, and although the expression may appear strange, it is, it seems to me, quite applicable. For illustration I will suppose that twenty practitioners had determined to watch the effects of the use of any new remedy; had agreed to adopt some particular plan of investigation, and to meet once a month, or oftener, for the purpose of comparing their experience and of discussing the results obtained. Suppose, in their practice, an equal number of cases to occur, and in one month we have almost the experience which a single practitioner would have gained in two entire years.

But this rapid experience, of as much consequence as it is, is the least important matter to be considered here—we have bearing upon a single subject, minds of a variety of casts, each of which must take a peculiar view; and the mere bringing of these views together, cannot fail to elicit something new and important.

It may be said here, that all this would be accomplished by means of a well conducted journal, to which all might submit their views, and the results of their experiments. If this could be done, it may readily be granted, that it would answer a most excellent purpose; but the objection that many—a great many—who would readily meet here, and on the spur of the occasion, speak freely of what had come under their notice, from want of time, inclination or habit, could not be induced to present their views in this manner, is insurmountable. Besides, it could not at all compensate for the loss of the new ideas which are always elicited by the verbal discussion of any subject.

A practice of this kind which will form a part of the plan of our association, will be the means, too, of inducing many to observe closely, who were before in the habit of observing carelessly, or, perhaps, not observing at all. It would, indeed, tend greatly to improve the habit in the most observant, for it could not fail to suggest ideas, to every one, which would increase his interest in his profession and widen the scope of his views.

Besides the great advantages which would result from association, with regard to points of this character, no one could fail to improve in the practical details of his profession. There are no two persons, as you all well know, who operate precisely alike, and from each other we will all learn something, which will have a tendency to improve our peculiar method. It must not be supposed, either, by gentlemen who have taken great care, and devoted much attention to the cultivation of their profession, that they are to give all, and gain nothing—from the humblest practitioner they may frequently learn what may be of great advantage to them. For myself, I am free to say, that I have never witnessed the most imperfect attempts, without observing some little thing which subsequently tended to enable me to operate more perfectly.

Besides these, a great many other important uses which might be effected by means of an association, present themselves to my mind, and will, probably suggest themselves to yours—to one I will briefly allude. An effort made by a body of this character, will be much more effectual in ridding the profession of the crowd of impostors which now encumber it. There are many ways, it is true, in which an individual may influence the public mind, to a considerable extent, and accomplish much good, but he will generally find himself unable to bring into operation any of those strong measures which, in some cases, (and the one before us seems to be peculiarly of that class,) are necessary for the suppression of injurious imposture. In a few years, if not at once, we may be able to bring legislative action to bear upon this matter, and so effectually curb, in the advances of such individuals, as those to whom we have been alluding.

And now, gentlemen, let me ask, if these are not objects worth striving to accomplish: the establishment of a warm and kindly social feeling amongst us, the furnishing of a medium through which we may impart our views and compare our experience upon commonly interesting subjects; by means of which we will improve in the practical details of our profession; and, lastly, to be enabled to take effective measures for the purpose of removing from our shoulders, the obloquy of being classed as members of the same profession with the ignorant and dishonest. Will a society, enabling us to accomplish these objects, be useless? I do not know how gentlemen present may feel with regard to the subject, but it is a source of great satisfaction to me, to know, that in however humble a degree, I have been, in some measure, instrumental in effecting its formation.

All that I have thus far said, has had reference to what we alone, as members of the dental profession, were to gain by means of this association. But it must be remembered, that in thus making ourselves more thoroughly acquainted with the principles, and skilled in the practice of our profession, we are rendering ourselves more useful to the community. Indeed, it is highly important, as I shall now attempt to show, that we should keep steadily in view a higher purpose than that of the mere advancement of our own selfish ends, in order that our efforts may be crowned with permanent success; and what higher object can we hope to attain, than by our united efforts to benefit the whole community. This will furnish us with a rule of action, without which, in many cases, it would be difficult indeed to move, and will contribute more towards the estab lishment of harmony and good feeling amongst us than any thing else.

I have said that our success in carrying out the objects of this association depends, in a great measure, upon the unselfishness of our ultimate ends, and although this may seem to some of you a strange position, I hope before I conclude, fully to establish it.

Reflect for a moment, if you please, upon the causes which have induced many to withhold themselves from us on this occasion. Why is it that they have refused to respond to our call? We have waited, and, as occasion offered, urged it upon those who might have been supposed to have most influence in this matter, and who, it might be believed, could command the respect and co-operation of all the worthy members of the profession, to make some movement in the matter, but in vain. Fully convinced that those who were older than ourselves would not be the first movers in the cause, a few of us who had long been satisfied of the importance, nay, the necessity, of the establishment of an association of dental surgeons, in this state, took the requisite preliminary steps for that purpose. In doing so, however, all concerned were extremely cautious to do nothing which would have the appearance of desiring, presumptuously, to thrust themselves conspicuously forward. They endeavored to show, in their efforts towards the accomplishment of this purpose, that their sole object was the formation of a society that they, and the community, might reap the advantages of association. They approached the older members of the profession in the most respectful manner, and begged them to come forward and aid them by their advice and influence. But they did not respond to the call. I will not attempt to say to you what I may suppose to be the motives which induced them to keep aloof—I will do nothing more than mention some of the reasons given by them for refusing to join in the movement, and leave you to draw your own inferences. Some said that they could not see how they were to be benefitted by joining a society, as they had, already, more practice than they could well attend to. Another would give his aid to no such movement, without all concerned were graduates in medicine. Others determined they would not associate themselves with those who charged less for their services than themselves. In all the refusals to co-operate with us, as far as I have been able to learn, reasons, such as these, were given.

And now let me ask, if those who were enjoying, at the hands of the community, a lucrative practice, had rightly considered their duty to the public, they would have refused to lend their aid to a movement which could not fail, ultimately, to benefit the whole community, because it did not promise to put more coin in their pockets? If those who considered a medical education necessary to constitute a competent dental surgeon, would not be better performing their duty towards the public, by assisting in a step which, in a few years, would make such education necessary to give the community confidence in the ability of the candidate for practice? And I would ask, again, if it would not have been better for those who refused to associate with us, because some concerned in the movement charged less for their services than themselves, if they supposed the objectionable individuals could not do well what they professed to do for so small a remuneration, for them to have joined in taking measures for so raising the standard of professional qualifications, that those engaged in the practice would find it absolutely necessary to perform their operations with so much care and labor, that they would be compelled, in order to make a livelihood, to increase their charge for their services.

I would willingly have avoided such allusions as these to any members of our profession, and should not have made the above remarks, except for the accomplishment of these two purposes: that some reason may be known, why, in this state, a movement has been made for the formation of a society of dental surgeons, by the younger members of the profession; and, of showing the danger to ourselves, now that we have entered into an association, of giving course to such feelings as seemed to have moved these gentlemen.

And we shall find, as we go on, great and frequent necessity of sacrificing our own selfish desires for the good of the community; but, in so doing, we shall become wiser, better and happier men.

But, as the time I had proposed to devote to this address is drawing to a close, and I cannot but feel that in extending it, although I felt great desire to do so, I should be taxing your patience too greatly, I shall only make some application of the principles I have advanced.

Our profession, as a profession, is yet in its infancy, and it must be a long time before a majority of those engaged in its practice can have many claims to its rights and privileges. The means of dental education have, till within a few years past, been very limited, and many who have desired to avail themselves of every facility for improvement, are still, although practitioners, very deficient in information with regard to the necessary theoretical basis, and the practical details of their profession, but are still honestly endeavoring to qualify themselves to become fully worthy to occupy their responsible position. With this class of dental practitioners, for some years to come, we shall often meet; and what must be our rule of action towards them? It is quite natural that those who have, at great labor and expense, acquired a knowledge of their profession, should feel some chagrin at seeing themselves classed by the public with such persons, and be disposed to say, "Sirs, you are attempting to practise an important profession, about which you know nothing, and I will have no fellowship with you."

This would be the natural prompting of a selfish spirit, and, if indulged in by members of our association, an application for membership, from such an individual, would be at once rejected. And now a proper appreciation of our duty to the community would tell us, that because we discountenanced these men, there are many unable to judge of their qualifications, who would employ them, and thereby sustain much injury; and that, instead of turning away from them, we should furnish them, as far as lies in our power, with the means of becoming useful members of society. There may be some of this character, who have been already admitted as members of this association, (as nearly all of us, as yet, remain ignorant of the qualifications of each other,) although I do not know it to be the case, nor pretend to think that all may not be as well or better qualified than myself; but if it is so, how necessary is it to our peace and harmony, that, in our intercourse with them, we should be influenced by more elevated feelings than those of mere self-aggrandizement.

It must not for a moment be supposed, that I am disposed to encourage the introduction into the profession of unqualified persons; against this it is our bounden duty, as well to ourselves as to the community, to turn our faces. The means of proper education are now easily attainable, and every one who does not take advantage of them, before he offers himself to the public as a candidate for practice, should be discountenanced. I speak of those, only, who are already in the profession, and who show, by their conduct, that want of opportunity is all that has prevented them from being eminently qualified—who honestly, as far as they know, endeavor to discharge their duties.

There is another class of practitioners, and it is a source of regret to me that it is so large, towards whom, if we perform our duty rigidly, it will be especially necessary to call in the aid of the sustaining principle I have been endeavoring to bring to your notice. I mean the class composed of those who wilfully impose upon public credulitiy, to the discredit of our profession, with which they are identified, and the great injury of the community. Towards such we must pursue no medium course. Every means, consistent with honor and justice, must be used to place them in their true light before the public. This is a duty—an unpleasant duty it is true, but from it we must not shrink—and to enable us to accomplish this work fully, it is not difficult to see how necessary it is to keep in view the public good. It is possible, indeed we cannot hope to escape the duty, that we shall be called upon to canvass freely the claims of many such to the distinctions of our body. Let no one shrink from furnishing evidence, when it is in his power to do so, which will enable us to detect imposition, and, as far as it is in our power, to discourage it.

How gladly would I say more—how sincerely have I wished for the powers of eloquence to have placed these subjects before you in the light they deserve. But what I have already said may suggest more than any length of time I might occupy would enable me to present to you. I have already occupied much of your time, and, with a few words more, will close.

We are, most of us, young—life is before us—we have entered our profession at a most favorable period—the means of rapid improvement are within our reach. Let us not fail to take advantage of them. Those who have preceded us have done much for the advancement of the profession. Let us do more—and we may be able to accomplish more, because our efforts are made in unison. And, gentlemen, let us never forget that our profession is one in which not only the comfort, but the health of the community is involved; and, above all, in our efforts to elevate the standard of our profession, let us keep constantly in mind, that the true way of gaining the respect and esteem of the community, is to render ourselves eminently useful to it.

